# Evaporation of alcohol droplets on surfaces in moist air

**DOI:** 10.1073/pnas.2302653120

**Published:** 2023-09-11

**Authors:** Lisong Yang, Amir A. Pahlavan, Howard A. Stone, Colin D. Bain

**Affiliations:** ^a^Department of Chemistry, Durham University, Durham DH1 3LE, UK; ^b^Department of Mechanical Engineering & Materials Science, Yale University, New Haven, CT 06511; ^c^Department of Mechanical and Aerospace Engineering, Princeton University, Princeton, NJ 08544

**Keywords:** droplet evaporation, condensation, Marangoni stress, rim instability, lubrication theory

## Abstract

Droplets of alcohol-based formulations are common in applications from sanitizing sprays to printing inks. However, our understanding of the drying dynamics of these droplets on surfaces and the influence of ambient humidity is still very limited. Here, we report the drying dynamics of picoliter droplets of isopropyl alcohol deposited on a surface under controlled humidity. Condensation of water vapor in the ambient environment onto alcohol droplets leads to unexpectedly complex drying behavior. As relative humidity (RH) increases, we observed a variety of phenomena including enhanced spreading, nonmonotonic changes in the drying time, the formation of pancake-like shapes that suppress the coffee-ring effect, and the formation of water-rich films around an alcohol-rich drop. We developed a lubrication model that accounts for the coupling between the flow field within the drop, the shape of the drop, and the vapor concentration field. The model reproduces many of the experimentally observed morphological and dynamic features, revealing the presence of unusually large spatial compositional gradients within the evaporating droplet and surface-tension-gradient-driven flows arising from water condensation/evaporation at the surface of the droplet. One unexpected feature from the simulation is that water can evaporate and condense concurrently in different parts of the drop, providing fundamental insights that simpler models based on average fluxes lack. We further observed rim instabilities at higher RH that are well-described by a model based on the Rayleigh–Plateau instability. Our findings have implications for the testing and use of alcohol-based disinfectant sprays and printing inks.

## Significance

The evaporation of an alcohol droplet in moist air is more complicated than it seems due to condensation. We present a combined experimental and theoretical study of the evaporation of picoliter sessile droplets (isopropyl alcohol) under controlled relative humidity (RH). We report a series of morphological changes, including the formation of a pancake-droplet that suppresses the coffee-ring effect. We developed a quantitative model that captures the essential physics of the problem and shows good agreement with experiment. One unexpected feature from simulation is that water can evaporate and condense concurrently in different parts of the drop, providing fundamental insight into drying dynamics. We show that large compositional variations exist within drops with profound implications in formulation applications.

In the fifth volume of his classic novel, À la Recherche du Temps Perdu, Marcel Proust wrote: “The only true voyage of discovery … would be not to visit strange lands but to possess other eyes (1).” In this paper, we bring “other eyes” to a seemingly simple question: How does a droplet of alcohol dry on a surface? This old question has re-emerged due to the widespread use of alcohol-based sanitizing sprays as a first line of defense against microbial and viral agents (2), and by the increasing use of alcohol-based inks as substitute solvents for methyl ethyl ketone in continuous inkjet printing (3).

The evaporation of an alcohol droplet in air is more complicated than it might at first seem because alcohols are hygroscopic and condense moisture from the ambient air into the droplet (4–9). Internal mixing due to hydrothermal waves tends to lead to a uniform composition (6, 7). However, recent studies using microliter droplets have demonstrated the presence of surface-tension-gradient-driven flows arising from gradients in composition (8, 9). Gravitational effects cannot be neglected in microliter droplets (10, 11) and the flow within the drop may break symmetry and become chaotic (8, 12). In the absence of humidity, the dynamics of evaporation of alcohol drops follows the well-established behavior of pure liquids on wettable surfaces (13–16), but the behavior in moist air is not well-captured by existing models due to a lack of understanding of the spatiotemporal evolution of the composition field during the lifetime of a drop. Such knowledge is essential to understand the efficacy of disinfecting sprays and to obtain a uniform deposit in inkjet printing applications, where the drop composition is a key factor (17).

Here, we present a combined experimental and theoretical study of the evaporation of picoliter droplets of isopropyl alcohol (IPA) on a hydrophilic surface as a function of the relative humidity (RH) of the environment. For such small drops, typical of the size used in inkjet printing, gravity can be neglected and the droplets remain azimuthally symmetric (with the exception of a rim instability noted later). These assumptions may not hold for the microliter drops that are more often studied in the literature (6–9, 12, 18). In our experiments, we report a series of morphological changes with increasing RH, many of which can be reproduced by our model. Our model shows that water can evaporate and condense concurrently in different parts of the drop, demonstrating that models based on average fluxes are likely to fail in reproducing drying dynamics. We argue that large compositional variations exist within drops—up to 50% volume fraction—with profound implications for the behavior of formulations. The drying behavior we document is exquisitely sensitive to the RH in humid environments, which suggests that there may be unpredictable and inconsistent performance in practical applications where the RH is not controlled.

## Results

### Experimental Results.

**Fig. 1.**
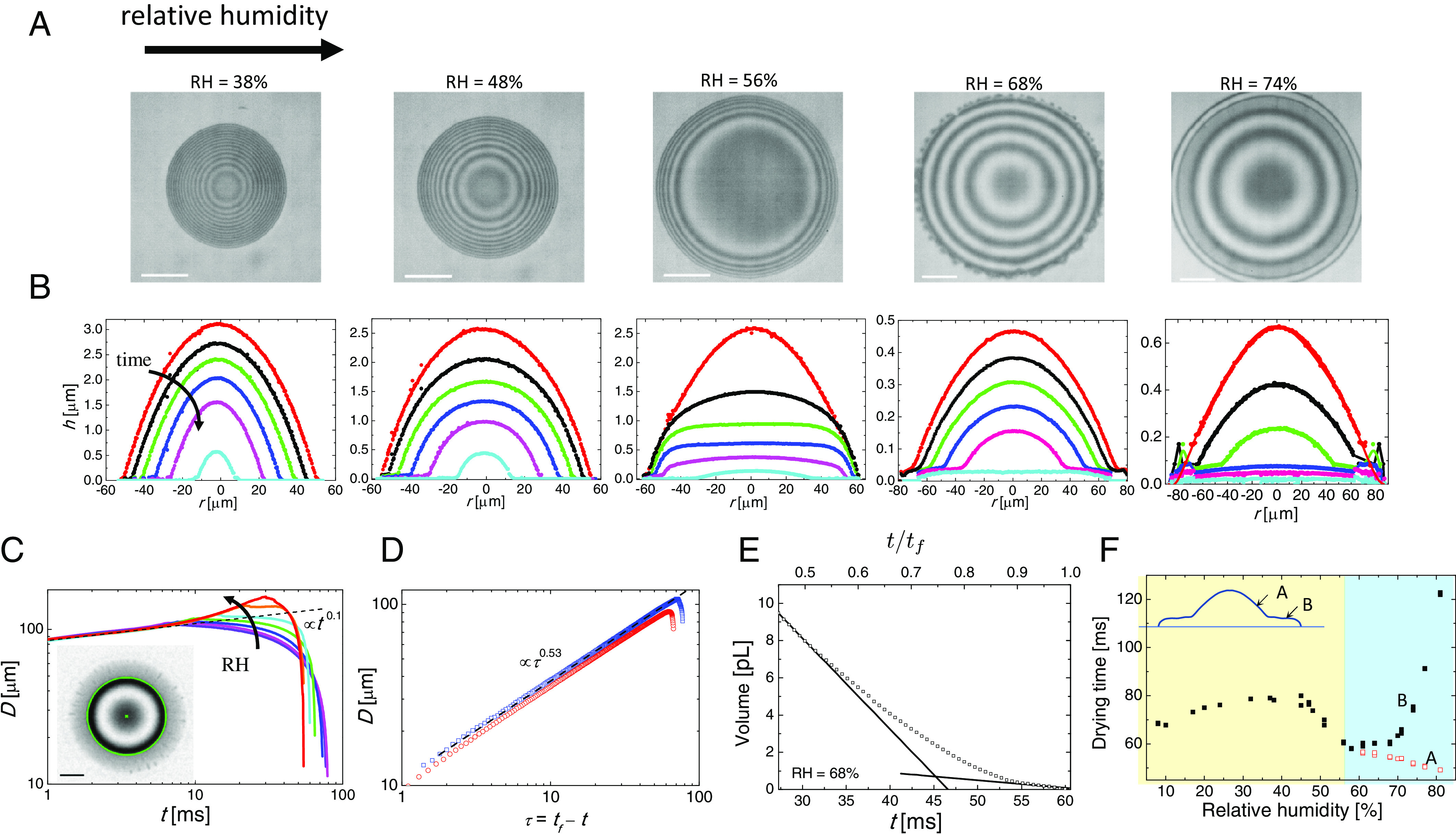
The evaporation dynamics of pure alcohol droplets changes significantly as the ambient RH increases. The initial droplet volume is 22 ± 2 pL. (*A*) Snapshots show interference fringes from droplets at half their lifetime (0.5 $t_{f}$) for RH of 38%, 48%, 56%, 68%, and 74%, from left to right. (*B*) The temporal evolution of droplet profiles in different ambient relative humidities corresponding to *A*. The times of the individual experimental curves for each humidity are as follows: from $0.2t_{f}-0.95t_{f}$ with a time interval of 0.15 $t_{f}$ for RH = 38%, 48%, and 56%; $0.65t_{f}-0.9t_{f}$ with a time interval of 0.05 $t_{f}$ for RH =68%; and $0.4t_{f}-0.9t_{f}$ with a time interval of 0.1 $t_{f}$ for 74%. Individual $t_{f}$ is shown in *F*. Droplet profiles at earlier times for RH = 68% and 74% are shown in *SI Appendix*, Fig. S1. (*C*) Contact diameter of a droplet as a function of elapsed time after impact for RH = 38%, 45%, 48%, 52%, 56%, 61%, and 68%. In cases where a residual water-rich film forms (RH = 61% and 68%), we plot the diameter of the central IPA-rich droplet. Inset: Snapshot of a droplet at 0.8 $t_{f}$ for RH = 68%. An IPA-rich droplet is defined inside the green circle, surrounded by a thin film (See also *F*, *Inset cartoon*). The black dashed line is the prediction of Tanner’s law. (*D*) Contact diameter as function of remaining time $\tau =t_{f}-t$ for RH = 10% (blue squares) and 38% (red circles). Black dashed lines are the fit to $D\propto \tau^{0.53}$ at the intermediate times. (*E*) Volume of a droplet as a function of time from $0.45t_{f}-1t_{f}$ for RH = 68%. The solid lines represent the theoretical estimates of the evaporation rates of a pure IPA droplet [−490 pL/s for a contact radius of 80 μm at $t/t_{f}=0.5$ (*Materials and Methods*)] and a pure water droplet [−41 pL/s for a radius of 70 μm at $t/t_{f}=0.9$]. (*F*) Humidity effects on droplet lifetime, $t_{f}$ (black solid squares), and, for RH > 60%, the lifetime of the IPA-rich central cap, $t_{f}^{\prime }$ (red open squares). *Inset*: Schematic of droplet shape with an IPA-rich cap, indicated as “A”, and water-rich brim, indicated as “B”, corresponding to *C*, *Inset*. All scale bars are 30 μm.

We deposit pure IPA droplets onto clean glass inside a humidity-controlled chamber. The droplet is generated from a 30-μm or 50-μm diameter inkjet nozzle with an initial droplet volume of 20–100 pL. The physical properties of IPA, water, and their mixtures are provided in *Materials and Methods*. We monitor the shape evolution of the deposited drop by high-speed interferometry (*Materials and Methods*). In interference images, the spacing of the fringes is proportional to the gradient in the film thickness; where there are no fringes, the surface is flat. Fig. 1*A* shows interferometric images of droplets halfway through drying ($t/t_{f}=0.5$ where $t_{f}$ is the drying time, i.e., droplet lifetime) and Fig. 1*B* shows the evolution of the reconstructed droplet shape (*Materials and Methods*). At RH < 40%, the droplet shape is a good approximation to a spherical cap; at intermediate RH (56%), the droplet turns into the shape of a pancake, while at higher RH (Fig. 1*A*, RH=68%) the roughly equal spacing of the fringes implies a conical shape. Later in drying at higher RH (e.g., RH = 68%, 74%), the shape evolves into a “wide-brimmed hat” with a dome surrounded by a flat brim. At RH > 70%, a raised rim forms around the edge of the drop and may break into tiny satellite drops. Drying droplets under a range of RH are demonstrated in Movies S1–S5.

Fig. 1*C* shows the evolution of the contact diameter, D, of droplets for a range of RHs. For the droplets that take the shape of a wide brimmed hat, we plot the diameter of the central (alcohol-rich) cap rather than the (water-rich) film around it (see below). For RH = 38%, the droplet spreads until ∼0.1$t_{f}$ and then retracts. For the ambient humidity above ∼45%, droplet spreading is significantly enhanced in time and space. The initial stage of spreading ($t/t_{f}<0.05$) is independent of RH and is well-described by Tanner’s Law (19), i.e., $D\propto t^{0.1}$. For RH < 45%, the whole drying process is qualitatively similar, with the droplet reaching its maximum diameter around $t/t_{f}=0.1$ and the retraction phase following the $D^{2}$-law (i.e., $D^{2}\propto \tau$, where the remaining time $\tau =t_{f}-t$, shown in Fig. 1*D*), in agreement with a diffusion-controlled theory (20); in other words, the alcohol droplets behave like pure liquids (see also *SI Appendix*, Fig. S2 for a different droplet size and substrate). For higher RH, the behavior deviates strongly from the behavior at low relative humidities, with the droplet continuing to spread as the RH increases and with the lifetime of the (central part of the) droplet decreasing.

The reconstructed droplet profiles can be used to calculate the volume of the residual droplet, V(t). Fig. 1*E* reports V(t) for RH=68% from $0.45t_{f}-1t_{f}$ (see *SI Appendix*, Fig. S1 for V−t curve for an extended time range). V(t) decreases linearly with a slope characteristic of the evaporation of pure IPA at $t/t_{f}=0.5$ (see the line in Fig. 1*E*) but at late times the evaporation rate is characteristic of that of pure water (see line at $t/t_{f}=0.9$). The formation of a water-rich film prolongs the lifetime of the droplet and leads to the nonmonotonic dependence of droplet lifetime on the RH with a minimum in the drying time at RH = 60% as shown in Fig. 1*F*. For RH > 60%, the lifetime of the central IPA-rich cap, $t_{f}^{\prime }$, continues to decrease with increasing RH while the lifetime of the droplet as a whole increases.

**Fig. 2.**
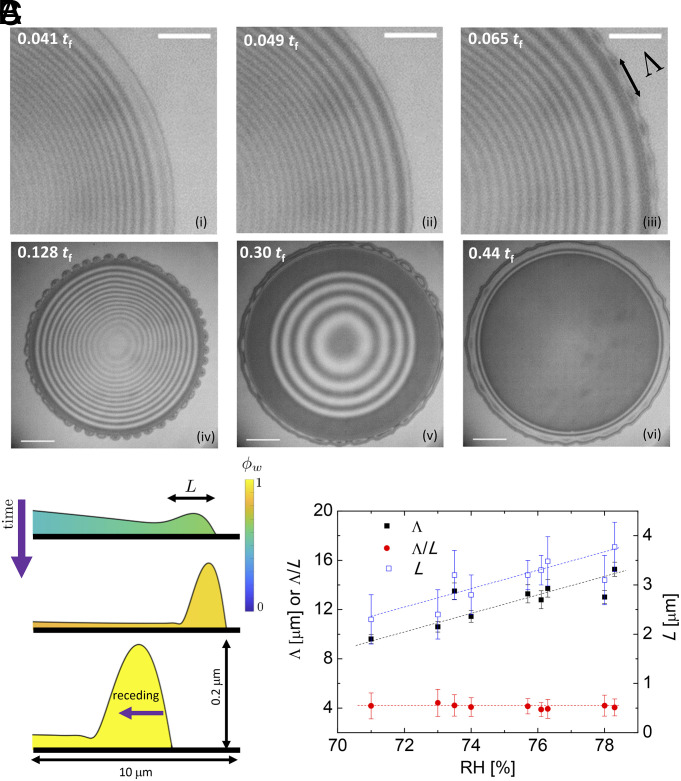
The edge of a droplet becomes unstable and forms finger-like protrusions at high RH. (*A*) Snapshots of a droplet at RH=78% at different times showing the development of the front instability: (*i*) A dimple forms almost in the middle of the foot, which has developed along the periphery of the droplet. (*ii*–*v*) The dimple region gets wider as the CL advances further and the IPA-rich droplet retracts. (*iii*) The periphery of the film is a rim where an instability appears and (*iv*) breaks into fingers. The instability wavelength Λ is labeled. (*v*) The contact line (CL) reaches its maximum extent. (*vi*) The alcohol-rich central cap evaporates. A ring forms near the CL. (Scale bar is 20 μm in (*i*–*iii*), and 50 μm in (*iv*–*vi*), respectively.) (*B*) The evolution of rim shape and composition obtained from the simulations for RH = 68%. (*C*) The instability wavelength Λ, the rim width L, and the ratio of these two quantities Λ/L as a function of the RH. Both the instability wavelength and rim width increase with RH while their ratio remains close to a constant value of Λ/L≈4. Dashed lines show the trend to guide the eye. The initial droplet volumes in this figure were 78 ± 4 pL and $t_{f}$ = 247 ms for the droplet in *A*. These droplets are larger than those displayed in Fig. 1 to show the rim instability more clearly.

For RH > 70%, we observe the formation of a raised rim around the edge of the drops, which becomes more pronounced as the RH increases. This rim is unstable and forms small finger-like protrusions shown in Fig. 2*A* (and Movie S6) for RH=78%. The contact line advanced to its maximum position at 0.3 $t_{f}$ and stayed still until the central IPA-rich cap disappeared at 0.44 $t_{f}$. During this time, fluid continuously fed into the fingers through a nanometrically thin film.

### Numerical Simulations and Comparison with Experiments.

**Fig. 3.**
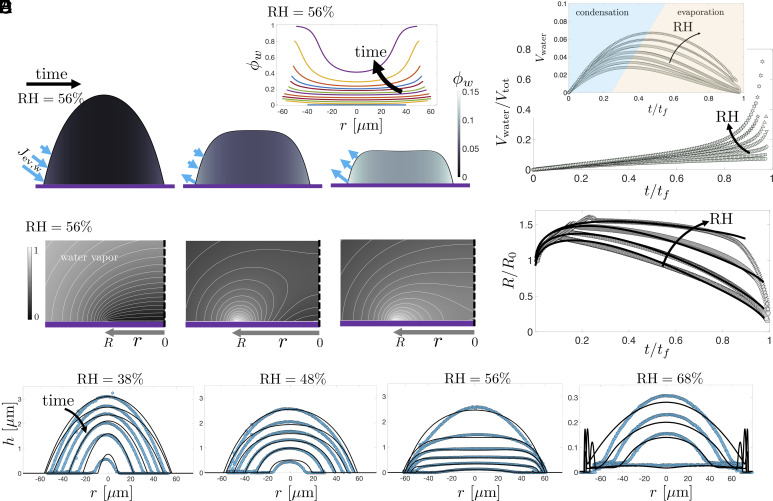
Our theoretical model predicts that water condenses into the alcohol droplet, creating compositional gradients and driving Marangoni flows, before evaporating. (*A* and *B*) The evolution of water volume fraction $\phi_{w}$ within a droplet at RH = 56%, showing a higher water concentration near the edge. At late times, the liquid at the edge tends to become pure water $\phi_{w}\approx 1$. (*C*) The water vapor field, corresponding to the snapshots in (*A*), demonstrates the spatiotemporal complexity of the evaporation flux with concurrent condensation and evaporation of water at intermediate times. (*D*) The volume of condensed water increases with the increase in ambient RH and the drop becomes water-rich at late times for high RH values, indicating the dramatic change in the composition of the drop as it evaporates. (*E*) Radius, R, of a droplet as a function of time after impact for various RH. Lines represent simulations and symbols represent experiments. In the experimental data, the droplet radius, R, is normalized by $R_{0}$, where $R_{0}$ is the radius in the frame immediately after the impact phase is over (*t*∼ 1 ms), and the time is normalized by $t_{f}$. (*F*) The temporal evolution of droplet profiles for different ambient relative humidities. Symbols represent the experimental data, and lines represent simulations. For the simulation, the initial volume was fixed at 20 pL and RH at 30%, 45%, 60%, and 70% from left to right plots.

To gain insight into the experimental observations, we model the flow field and the shape of the drop using the lubrication approximation coupled with a quasi-steady diffusion-limited vapor field (21). We consider the nonideality of the mixture to determine the vapor concentration on the interface of the drop (22). The model is described in *Materials and Methods*. Simulations reveal the development of spatial composition gradients within the alcohol drops as water condenses (Fig. 3 *A* and *B*). At very early times, water condensation is strongest at the edge of the drop. The condensation of water at the edge drives a solutal Marangoni flow from the alcohol-rich center toward the edge. This accumulation of water at the edge increases the local water vapor concentration at the edge and switches the condensation to evaporation. At intermediate times, water continues to condense in the middle of the drop while evaporating at the edge (Fig. 3*C*, *Middle*). Once the water saturation vapor pressure everywhere in the drop exceeds the ambient vapor pressure, condensation ceases altogether and water evaporates (Fig. 3*C*, *Right* and *D*, *Inset*).

Increasing the ambient RH increases the amount of water condensed into the alcohol drop and at high enough RH the drop becomes nearly pure water (Fig. 3*D*). The Marangoni flows arising from the compositional gradients affect the spreading and drying dynamics (Fig. 3*E*) as well as the shape of the drops (Fig. 3*F*), leading to the formation of flattened pancake-like shapes at higher RH values, and rims at the contact line. At RH > 60%, the quantitative agreement between the simulations and experiments deteriorates, which may be a consequence of the breakdown of the vertical-mixing assumption or water condensation at the nozzle (see *Discussion*, *Materials and Methods*, and *SI Appendix*), but the qualitative features are still reproduced by the model (Fig. 3*F*). We note that models that invoke an evaporative flux based on the average droplet composition do not agree with experiments; it is necessary to solve the Laplace equation in the vapor with a spatially varying boundary condition at the liquid–air interface (23, 24).

## Discussion

Our study has been carried out with droplets of a size typically used in inkjet printing. Our theoretical model, however, is scale-independent and therefore the predictions will hold for larger droplets (such as nanoliter drops typically found in sprays) provided that gravitational and thermal effects remain negligible and the droplet remains azimuthally symmetric. In *SI Appendix*, Figs. S2 and S3, we present data for somewhat larger droplets (80 pL compared to 20 pL), which show the same drying behavior for both drop sizes at a range of RHs. We show that the drying behavior is the same on glass and sapphire substrates, which have very different thermal conductivities (*Materials and Methods*) suggesting that thermal effects are negligible. Law previously reported insignificant thermal effects on the evaporation of IPA drops suspended in moist air (4). Thermal effects have been reported with more volatile liquids, such as ethanol or methanol sessile drops or hydrofluoroethers drops in spray (6, 7, 25). Thermal Marangoni-induced instabilities can cause the breaking of axial symmetry in large drops (12), but we did not observe such instabilities with pL drops of IPA. We provide a more detailed discussion of thermal effects in *SI Appendix*.

Care was taken in the handling and storage of IPA to minimize absorption of water from the atmosphere. Water absorption also occurs in the nozzle before printing. The extent of water absorption is discussed quantitatively in *SI Appendix*, where we also present experimental evidence to show that water absorption in the nozzle does not change the qualitative drying behavior.

Toluene has a similar evaporation rate to IPA but is mutually insoluble with water (water solubility in toluene is 0.033% at 25${}^{\;{}^{\circ}}$C and water is not surface active). Moist air shows no obvious effect on the toluene droplet dynamics and no water-rich film is left behind a toluene droplet at high RH (*SI Appendix*, Fig. S7), showing that the effects of evaporative cooling and vapor condensation on the substrate are negligible in our study.

The rim instability that we observed is reminiscent of other types of fingering, including gravity-driven (26, 27), thermal Marangoni (28, 29), solutal Marangoni (30, 31), or the instability of a dewetting front (32). Our simulation shows that the rim forms due to the forced spreading under solutal Marangoni stresses from the IPA-rich center toward the water-rich edge at early times (Fig. 2*B*). We characterize this instability by defining the wavelength of instability as $\Lambda =2\pi R_{\mathrm{rim}}/N$, where $R_{\mathrm{rim}}$ is measured from the center of the droplet to the middle of the rim and N is the number of beads identified at the early stage of instability (Fig. 2*A*). We find that the ratio of the wavelength of instability Λ to the width of the rim L, i.e., Λ/L=4.1±0.2 (Fig. 2*C*). Brochard-Wyart and Redon showed that the fastest growth rate of the Rayleigh–Plateau instability of a ridge of liquid on a solid satisfies $q_{m}L$ = 1.5, where $q_{m}=2\pi /\Lambda$ is the wave vector, i.e., Λ/L=4.2 (33), in good agreement with the experimental results.

The physicochemical hydrodynamics of the evaporation of multicomponent drops is a topical field of research (34–37). Droplets from a mixture can undergo enhanced or suppressed spreading induced by the differences in volatilities and surface tensions between the components, as well as the nonuniform evaporation flux across the droplet (8, 21, 38–42). In this paper, we have shown that even the drying of a simple alcohol is a complex process, due to moisture condensation, and which exhibits rich fluid-dynamical features. Our results show that the surface area covered by a single drop, the composition within the drop, and the time that the droplet remains above a threshold alcohol concentration are all strongly dependent on the RH. While an alcohol-based disinfectant with a typical alcohol concentration of 70% in water will behave differently from a pure alcohol, differential evaporation of water and alcohol will still cause variations in morphology and composition that depend strongly on the RH. Similarly, the drying behavior of a single droplet will not be identical to that of a distribution of many droplets on a surface, but the common physics means that strong dependence on RH is likely. This complexity has practical consequences. The efficacy of alcohols in sanitizing applications depends on the water content of the alcohol and the duration of the exposure of the pathogen to the solution (43). Hence the efficacy of a sanitizing spray measured at one humidity cannot necessarily be extrapolated to other humidities. This conclusion has ramifications for testing regimes of alcohol-based sanitizers, which has not been reported in the public health sector, to our knowledge.

We have also shown that the morphology of evaporating droplets is highly sensitive to RH. In our previous work (21, 42), we demonstrated that the formation of pancake-like shapes in alcohol blends can mitigate the coffee-ring effect and we observe the same outcome for the pancake-shaped drops here (see *SI Appendix*, Fig. S9 and Movies S7–S9). We anticipate, therefore, that the control of RH will be an important factor for creating uniform thin films from alcohol-based inks. In printing, the solvent composition of the ink is chosen to form a stable suspension of pigments or nanoparticles. The large spatial variations we observe in the water content of alcohol-based inks may therefore lead to selective aggregation or precipitation of colloidal particles and nonuniform deposits.

## Materials and Methods

### Experimental Protocol.

A schematic of our experimental setup is presented elsewhere (21). In brief, droplets of a typical volume of 20–100 pL are dispensed from a drop-on-demand device (MJ-ABP-01, MicroFab Technologies), with a 30-μm or 50-μm orifice, through a driver (JetDrive III controller CT-M3-02). A light-emitting diode (470 nm, Thorlabs) illuminates the sample from underneath the substrate and the reflected light is collimated, passed through a bandpass filter (bandwidth 10 ± 2 nm, Thorlabs, to control the coherence length), and imaged onto a high-speed camera (Photron, SA4). The reflected light from the liquid–air and liquid–solid interfaces interferes and fringes can be observed when the thickness of the film is less than the coherence length of the light source. A shadowgraph from the side of the sessile droplet, recorded with a high-speed camera (Optronis, CR450x3) through a telescope (Navitar x12 zoom), is used to determine the initial droplet volume and shape. The two cameras and MicroFab controller are synchronized to the trigger signal from the Photron camera. The imaging system is calibrated with a microcalibration plate (Lavision).

A humidity control line and cell are designed to control the ambient RH of the drying droplet. Nitrogen is delivered to a flow splitter via a pressure gauge into two flowmeters (Cole-Parmer, maximum flow rate of 100 mL/min for air). One flow stream from a flowmeter is fed into a bubbler with a porous frit to generate bubbles containing the saturated water vapor. The other flow stream through the second flowmeter contains dry nitrogen. The dry and wet nitrogen gases are brought into a mixer. The mixture is then fed into a humidity cell (40 mm x 40 mm x 15 mm) where the flow runs a few millimeters below the sample to avoid disturbing the drying droplet. The temperature and RH evaluation kit (Sensirion EK-H5 with sensor SHT31) with temperature accuracy of ± 0.3${}^{\;{}^{\circ}}$C and humidity of ±2% is fitted into the cell. The sensor was calibrated with twelve salts covering the range of RH from 6.6 to 94.6% with the expected RH values taken from literature (44). By adjusting the dry/wet nitrogen flow, an RH ranging from 5 – 85% is obtained with a fluctuation of 0.2%. The flow rate is minimized for each RH. Temperature is typically 22.0 ± 0.5${}^{\;{}^{\circ}}$C inside the humidity cell. The MicroFab nozzle is 1–2 mm above the substrate inside the humidity cell. The typical nozzle idle time is 2 s before the generation of a single (or a train of) droplet(s) on a fresh area of a substrate for recording.

The contact line of the droplet is traced and analyzed as a function of time, and the droplet shape is reconstructed from interference fringes via a custom-written Matlab code. Two neighboring bright or dark fringes traced either in space or time have a thickness difference of $d_{\lambda }=\lambda /\left(2n\right)∼$ 170 nm, where n is 1.383, the refractive index of IPA at wavelength, λ, of 470 nm. To determine the film thickness h between neighboring bright and dark fringes, we use a local fit of a sinusoidal function of I(h) by $I\left(h\right)=I_{0}+A$cos$\left[2\pi \left(h-h_{1}\right)/d_{\lambda }\right]$, where $I_{0}=\left(I_{1}+I_{2}\right)/2$, $A=(I_{1}-I_{2})/2$, $I_{1}$ and $I_{2}$ are the local maximum and minimum intensities for film thickness $h_{1}$ and $h_{2}$, respectively. The volume of a droplet is calculated mainly from the profile assuming the droplet to be axisymmetric, or at early times, from the side-view image. The error in the volume arises from the uncertainty in radial position and thickness. The uncertainty in radial position in an image is ∼1 pixel, i.e., 0.4 μm, and the uncertainty in thickness from interferometry is ∼5 nm. We do not correct the thickness for the time-varying refractive index (RI) of IPA–water mixtures. The RI of IPA-rich mixtures is relatively insensitive to composition since the reduction in RI due to the mixing with water (through the Lorentz–Lorenz equation) is compensated by the increase in RI due to the negative excess volume of mixing (through the Clausius–Mossotti equation) (45). For volume fractions of IPA > 45%, the error in thickness due to the assumption of a constant RI is < 1%.

IPA was purchased from Fisher Scientific (≥99.5%, Reagent Grade, 1 L). The storage and handling of IPA is described in *SI Appendix*. Poly(N-vinylpyrrolidone) (PVP)-stabilized polystyrene (PS) particles (755 nm) are used as the tracer particles for the deposition patterns. Dry PVP-PS particles are weighed to make suspensions in a range of 0.04–0.06 wt% through 30-min bath sonication. The suspensions exhibit no obvious sedimentation over a day.

Microscopic glass coverslips (Academy Science) with a thickness of 0.13–0.17 mm and sapphire coverslips (UQG Ltd optics) with a thickness of 0.17 mm were cleaned by the following procedure: first, soak in 5 wt% Decon 90 with sonication (heat up to 45${}^{\;{}^{\circ}}$C) for 30 min; then rinse with ultrapure water (Elga, Chorus 1 Analytical Research) and soak in IPA overnight; blow dry with N${}_{2}$ and plasma clean in air (Bio-RAD, Plasma Asher E2000) for 15 min; finally, rinse with ultrapure water and dry with N${}_{2}$. All samples were prepared under laboratory ambient conditions: RH=45±5% and T=21±1${}^{\;{}^{\circ}}$C, and used on the day they were prepared.

Both glass and sapphire substrates are hydrophilic with high surface free energies (46–48). At room temperature, sapphire has a much higher thermal conductivity of 46 W/m/K than that of glass of 0.96 W/m/K (49). The surface tension of a mixture is taken from literature with interpolation at a temperature of 22${}^{\;{}^{\circ}}$C (50). Physical properties of pure water and IPA are listed in Table 1.

**Table 1.** Physical properties of water and IPA

Solvents	$p_{s}$/kPa*	$D_{v}\times 10^{-5}$/m${}^{2}$s${}^{-1}$†	$M_{w}$ g mol${}^{-1}$	σ/mN m${}^{-1}$‡	μ/mPa s	ρ/kg m${}^{-3}$
Water	2.62	2.58	18	72.5	1.0 (25 (${}^{\;{}^{\circ}}$C)	998 (${}^{\;{}^{\circ}}$C)
IPA	5.0	0.98	60	21.5	2.3 (25 (${}^{\;{}^{\circ}}$C)	786 (20${}^{\;{}^{\circ}}$C)

$p_{s}$: saturated vapor pressure; $D_{v}$: diffusion coefficient in air; $M_{w}$: molecular weight; σ: surface tension; μ: viscosity (51); ρ: liquid density (52). The temperature is 22${}^{\;{}^{\circ}}$C unless otherwise stated.

^*^$p_{s}$ is calculated from the Antoine equation ($log_{10}p_{v}=A+B/T+Clog_{10}T+DT+ET^{2})$, where T is the absolute temperature and $p_{V}$ is in mmHg in ref. 51 and converted to kPa in the table (51).

^†^Diffusion coefficient is calculated from $D_{298}=D_{v}{\left(298/T\right)}^{2}\left(p/760\right)$ where $D_{298}$ is the value taken at 25${}^{\;{}^{\circ}}$C (53).

^‡^σ is calculated by linear interpolation between values at 25${}^{\;{}^{\circ}}$C and 20${}^{\;{}^{\circ}}$C (50).

We estimate the evaporation rate of a droplet of pure liquid, i.e., IPA or water, of volume V as $dV/dt≃-4RD_{v,i}c_{s,i}\left(1-H_{i}\right)/\rho_{i}$, where i=a,w stands for IPA and water, respectively, $H_{i}$ is the relative vapor density (or humidity for water, i.e., RH), R is the droplet contact radius, $D_{v,i}$ is the diffusion coefficient of the vapor, $c_{s,i}=M_{w,i}p_{s,i}/\left(R_{\mathrm{gas}}T\right)$ is the saturated vapor density according to the ideal gas law, $M_{w,i}$ is the molar mass, $R_{\mathrm{gas}}$ is the gas constant and $p_{s,i}$ is the saturated vapor pressure (54). For pure IPA the ambient vapor density $c_{v,a}{|}_{r\to \infty }$ = 0, i.e., $H_{a}=0$, whereas the ambient RH $H_{w}$ = RH = $c_{v,w}{|}_{r\to \infty }/c_{s,w}$.

The average water condensation rate onto an IPA/water mixture is proportional to $p_{s,w}$RH$-p_{w}$, where $p_{w}$ is the partial vapor pressure of water in the mixture, i.e., $p_{w}=\Gamma_{w}x_{w}p_{s,w}$ and $\Gamma_{w}$ and $x_{w}$ are the water activity coefficient and molar fraction, respectively. The critical RH can be determined by $\Gamma_{w}x_{w}$, above which water condenses into the mixture (*SI Appendix*, Fig. S8).

### Theoretical Model.

We model the flow within the drop using the lubrication approximation given that the height of the droplets $h_{0}\approx$ 1 μm is much smaller than their radius R≈ 50 μm, i.e., $h_{0}/R\ll 1$, and inertial effects are negligible compared to the viscous effects, i.e., the Reynolds number is small, where $\text{Re}=\rho Uh_{0}\left(h_{0}/R\right)/\mu ∼10^{-4}$, with U=O(1) mm s${}^{-1}$ as the characteristic liquid velocity and μ≈ 2 mPa.s and $\rho \approx 800\;\text{kg}{\text{m}}^{-3}$ as the liquid viscosity and density, respectively. Gravitational effects are also negligible given that the Archimedes number $\text{Ar}=gh^{3}\rho_{0}\Delta \rho /\mu^{2}\approx 10^{-9}\ll 1$, where $\rho_{0}$ and Δρ are average and difference density of water and IPA, respectively (11). The timescale of diffusion of the components across the height of the drop scales as $h_{0}^{2}/D_{l}\approx$ 1 ms, where the liquid diffusivity $D_{l}\approx 10^{-9}\;{\text{m}}^{2}{\text{s}}^{-1}$. This diffusion timescale is much smaller than the evaporative timescale of the drop $h_{0}/\left(J_{\mathrm{ave}}/\rho \right)\approx$ 25 ms, where $J_{\mathrm{ave}}$ is the average evaporative flux for a pure IPA droplet; this indicates the components are well-mixed across the height of the drop. We treat the drop as axisymmetric and the model cannot therefore treat the rim instability in Fig. 2.

Therefore, the coupled evolution of the height of the drop h(r,t), and the volume fractions $\phi_{i}\left(r,t\right)$ with i = a, w representing the two volatile components, i.e., IPA and water, respectively, are described by

[1] $$\begin{array}{c} \frac{\partial (h\phi_{i})}{\partial t}=\underset{\mathrm{diffusion}}{\underbrace{\frac{1}{r}\frac{\partial }{\partial r}\left(rhD_{l}\frac{\partial \phi_{i}}{\partial r}\right)}}-\underset{\mathrm{convection}}{\underbrace{\frac{1}{r}\frac{\partial }{\partial r}\left(rh\overset{¯}{u}\phi_{i}\right)}}-\underset{\mathrm{evaporation}}{\underbrace{J_{ev,i}}}, \end{array}$$

in which $J_{ev,i}$ are the evaporative fluxes of the two components. The height-averaged velocity is defined as

[2] $$\begin{array}{c} \overset{¯}{u}\left(r,t\right)=-\underset{{\overset{¯}{\text{u}}}_{\text{Ca}}\text{: capillary flow}}{\underbrace{\frac{h^{2}}{3\mu }\frac{\partial p}{\partial r}}}-\underset{{\overset{¯}{\text{u}}}_{\text{Ma}}\text{: solutal Marangoni flow}}{\underbrace{\frac{h}{2\mu }\frac{\partial \gamma }{\partial \phi_{a}}\frac{\partial \phi_{a}}{\partial r}}}, \end{array}$$

where the liquid pressure p=γκ+Π(h) with $\Pi \left(h\right)=A/h^{3}$ as the disjoining pressure, $A\approx 10^{-20}$ J as the Hamaker constant, and the curvature $\kappa =-\nabla^{2}h$. We denote $\phi \equiv \phi_{a}$ as the volume fraction of the more volatile IPA, and the volume fraction of water is $\phi_{w}=1-\phi$. The second term in Eq. **2** represents the contribution of the solutal Marangoni flow due to gradients in the surface tension γ(r,t), which is a nonlinear function of the volume fraction. In experiments and simulations, the radial Peclet number, Pe${}_{r}=UR/D_{l}\approx 100$, where U is the radial flow speed ∼10${}^{-3}\;{\text{ms}}^{-1}$, R is the contact radius and $D_{l}$ is the mutual diffusion coefficient of the liquid. The vertical Peclet number, Pe${}_{v}=\epsilon^{2}$-pagination Pe${}_{r}$ where the aspect ratio of a droplet, $\epsilon =h_{0}/R_{0}\approx 0.01$; so Pe${}_{v}\approx 10^{-2}$. We therefore do not correct the mutual diffusion coefficient for shear dispersion.

The characteristic timescale of vapor diffusion $\tau_{D}=R^{2}/D_{v}\approx 1$ ms, where $D_{v}\approx 10^{-5}{\text{m}}^{2}{\text{s}}^{-1}$ is the vapor diffusion coefficient, is much smaller than the evaporative timescale of the drop evaporation $t_{f}\approx 25$ ms. Thus, we model the vapor field as diffusion-limited and quasi-steady: $\nabla^{2}c_{v,i}=0$, where $c_{v,i}$ are the vapor concentrations of the two components. A thin drop can be approximated as a disk with zero height. For a nonideal mixture, the vapor concentration on the interface of the drop is proportional to the saturation concentration $c_{v,i}=\Gamma_{i}x_{i}c_{s,i}$ with the activity coefficient $\Gamma_{i}$, and mole fraction $x_{i}=\left(\phi_{i}/M_{i}\right)/\left[\phi /M_{a}+\left(1-\phi \right)/M_{w}\right]$ ignoring the density difference between the two liquids, where $M_{i}$ are the molecular weights of the two components. The partial vapor pressure of IPA–water mixture is derived from activity coefficient data (22) (*SI Appendix*, Fig. S8). The evaporative fluxes are $J_{ev,i}=-\left(D_{v}/\rho \right)n\cdot \nabla c_{v,i}$, where n is the unit normal vector directed away from the liquid phase.

## Supplementary Material

Appendix 01 (PDF)

Movie S1. IPA droplet drying on glass under RH of 38%.

Movie S2. IPA droplet drying on glass under RH of 56%.

Movie S3. IPA droplet drying on glass under RH of 61%.

Movie S4. IPA droplet drying on glass under RH of 68%.

Movie S5. IPA droplet drying on glass under RH of 74%.

Movie S6. IPA droplet drying on glass under RH of 78%.

Movie S7. IPA droplet drying with trace particles on glass under RH of 46%.

Movie S8. IPA droplet drying with trace particles on glass under RH of 54%.

Movie S9. IPA droplet drying with trace particles on glass under RH of 64%.

Movie S10. Drying of two successive IPA droplets on glass under RH of 74% with idle times of 2 s for the first droplet and 0.5 s for the second droplet.

## Data Availability

The raw experimental data associated with the data in the figures are available through the University of Durham data repository (https://doi.org/10.15128/r12801pg44n) (55).
