# Supplementary material for: Evaporation of alcohol droplets on surfaces in moist air
Source: Proc Natl Acad Sci U S A. 2023 Sep 11;120(38):e2302653120. doi: 10.1073/pnas.2302653120 (PMC10515150; doi:10.1073/pnas.2302653120)
Supplement: Supplementary file 1 — Appendix 01 (PDF) [file pnas.2302653120.sapp.pdf]

Supplementary Information for

Evaporation of alcohol droplets on surfaces in moist air

Lisong Yang, Amir A. Pahlavan, Howard A. Stone and Colin D. Bain

Colin D. Bain
E-mail: c.d.bain@durham.ac.uk

This PDF file includes:

Supplementary text
Figs. S1 to S9
Legends for Movies S1 to S10
SI References

Other supplementary materials for this manuscript include the following:

Movies S1 to S10

Supporting Information Text

Thermal effect in drying IPA droplet

In Figures S2 and S3, we show the drying behavior on two substrates - glass and sapphire - which have very different thermal conductivities (sapphire: 46 $\mathrm{W\,m^{-1}\,K^{-1}}$; glass: 0.96 $\mathrm{W\,m^{-1}\,K^{-1}}$ (1)). The drying behavior is the same on both substrates, showing that the substrate behaves as an isothermal heat source; there is no cooling of the substrate that leads to observable thermal effects in the drying of the droplets.

We have estimated the magnitude of thermal cooling of a pure IPA droplet in an earlier paper (2). The average rate of heat loss in a droplet due to the latent heat of evaporation is $J_{avg}\Delta H_v$, where the average evaporative mass flux $J_{avg} = 4RD_vc_a/(\pi R^2) \approx 0.03\ \mathrm{kg\,m^{-2}\,s^{-1}}$ with vapour density $c_a \approx 0.12\ \mathrm{kg\,m^{-3}}$ and $R = 50\ \mu$m, and the latent heat of evaporation per unit mass of liquid $\Delta H_v = 756$ $\mathrm{kJ\,kg^{-1}}$ (3). Under quasi-steady conditions, evaporative heat loss is balanced by heat conduction from the substrate (assumed to be isothermal, see above), i.e. $J_{avg}\Delta H_v \approx k_L\Delta T/h$, where h is the thickness of the drop at the apex $\sim 3\ \mu$m and the thermal conductivity $k_L = 0.14\ \mathrm{W\,m^{-1}\,K^{-1}}$ (25 °C, (3)). The temperature difference between the bottom and top of the drop, $\Delta T \approx J_{avg}\Delta_v Hh/k_L \approx$ 0.5 K (colder at the top). The ratio of the substrate to liquid thermal conductivities $k_R = k_S/k_L > 2$, so the drop is coldest at the apex (4). Consequently, the thermal Marangoni effect opposes spreading. Since Marangoni flows due to composition gradients enhance spreading under the conditions studied here, any thermal Marangoni effects would act to reduce the rate of enhanced spreading. The ratio of flow speeds from thermal Marangoni effects and evaporation is given (5) by the thermal Marangoni number $\mathrm{Ma}_T = -(d\sigma/dT)\Delta Tht_f/(R^2\mu) \approx 1$, where $d\sigma/dT = -0.1\ \mathrm{mN\,m^{-1}\,K^{-1}}$ (6)). Ma_T is not negligible, so we cannot a priori rule out thermal Marangoni flows making an observable contribution to the spreading dynamics. We note that studies on microlitre droplets have found that convection within drops typically leads to thermal Marangoni flow being $10-100$ times weaker than predicted using scaling arguments (7, 8). Our experiments show, however, that pure IPA evaporation displays the canonical spreading behavior for pure droplets on both glass and sapphire at both low RH (Fig. S2) and high RH (Fig. S3). We note that water condensation reduces thermal gradients in the droplet, so the largest thermal Marangoni effects would be expected in pure IPA. The good agreement between our experiments and simulations, which do not account for thermal effects, provides additional evidence that thermal effects in our system are weak compared to solutal effects.

Condensation effect at the nozzle

The initial condition for an IPA droplet printed onto a glass substrate is not pure IPA due to absorption of water in the nozzle and, to a lesser extent, in flight. To provide a quantitative estimate of the effect of water absorption in the nozzle on the initial droplet composition, we treat the nozzle for simplicity as a cylindrical channel with a radius of 15 μm and a constant, uniform flow of pure IPA from a reservoir towards the nozzle plate to maintain the meniscus at a constant position as the IPA evaporates. We can then write down a steady-state 1-D solution to the convective-diffusion equation in the nozzle in which the liquid at the nozzle surface is in equilibrium with the vapor at the nozzle. Figure S8c shows the equilibrium volume fraction of IPA for different RH. For example, for RH = 60%, the liquid at the nozzle surface has composition (by volume) of 92% IPA and 8% water. The water concentration then decays exponentially into the liquid with a decay length given by D_l/E, where $D_l = 6 \times 10^{-10}\ \mathrm{m^2\,s^{-1}}$ is the mutual diffusion coefficient in the liquid (9) and E is the flow rate in the nozzle (which is equal to the evaporation rate of the IPA, since at steady-state

there is no net condensation or evaporation of water). For a 92% IPA solution in a 15-μm diameter nozzle, $E = 120\mu\text{m s}^{-1}$, giving a diffusion length of 5 μm. The characteristic time to establish a steady-state profile is given by $D/E^2 = 40$ ms. Given that the delay before printing a drop is $\gg 40$ ms, we can assume that the composition profile in the nozzle is close to steady-state. Again assuming a cylindrical nozzle for simplicity and plug flow during the droplet formation process, a 22-pL drop is formed from the 17-μm depth of liquid nearest the nozzle, the average composition of which is 2.4% water and 97.6% IPA. This is only an approximate calculation of the initial composition of the droplet because the nozzle is actually tapered (which will slightly increase the water concentration in the drop) and the flow velocity is non-uniform due to the non-slip boundary condition on the walls of the nozzle (which will decrease the water concentration in the drop). This estimate also neglects Marangoni effects in the nozzle.

To explore the effects of water condensation on the experimental results we carried out two control experiments. First, we compared the drying behaviour of IPA droplets printed after a delay of 500 ms and 2 s (See Fig. S4 and S5). We would expect on the basis of the scaling argument above that the initial droplet composition should be the same for both of these time delays and we do indeed observe nearly identical behaviour. This control experiment is also important in showing that we do not have to control the delay before printing a droplet with great precision.

Second, we studied the drying behaviour of a droplet with an initial (reservoir) composition of 95% IPA by volume – a solution that contains more water than the calculation above yields for pure IPA in the nozzle at all the RH that we studied, shown in Fig. S6. At very low RH, the 95% IPA droplet shows qualitatively different behaviour from pure IPA due to Marangoni contraction (10), because the water is evaporating faster than the IPA (which in turn arises from a combination of the well-known non-ideality of mixing of water and alcohol (11) and the higher vapor diffusion coefficient of water). Marangoni contraction is not observed with pure IPA since at low RH there is negligible water condensation in the nozzle. At RH = 61% we observe the same qualitative features as for pure IPA. We conclude that water condensation in the nozzle does not materially affect the behaviour of IPA droplets over the range of RH reported in this paper.

Storage and handling of IPA samples

The master bottle from the purchase is poured to fill 2/3 of a 100-mL borosilicate glass reagent bottle inside a fume hood. The master bottle is resealed immediately and used for analytical purposes within a month after being opened. The secondary bottle is used for the experiment within two days. Beyond these times, bottles are labeled as cleaning solvent for general laboratory use. All bottles are stored in a cupboard for flammable liquids. Before experiments, a 5-mL IPA sample is carefully poured from the 100 mL bottle into a printing reservoir – a glass vial with a capacity of 7 mL. The filling process takes less than one minute. The bottles and vial are tightly closed immediately after filling. The vial lid is connected to the printhead via a micro Teflon tubing. The IPA in the reservoir is pressurized with dry air to control the meniscus in the nozzle. The printhead is primed for 10 minutes at a printing frequency of 1 kHz. We run experiments with a full range of RH (typical from 10% to 70%) within a day for a given nozzle size and substrate. The bottle and vial that are used here are pre-cleaned and dried by the following procedure: first, soak in 2 wt% alkaline detergent solution (Decon 90; Decon Laboratories) with sonication (heat up to 45 °C) for 30 min; fill and rinse with warm tap water twice and ultrapure water (Elga, Chorus 1 Analytical Research) twice, fill the bottle with washing IPA and soak overnight; finally, empty the glassware and blow dry with N_2 before use. Tubing and nozzle are generally cleaned by soaking in cleaning alcohol, sonication, and then blow dry with N_2 before use.

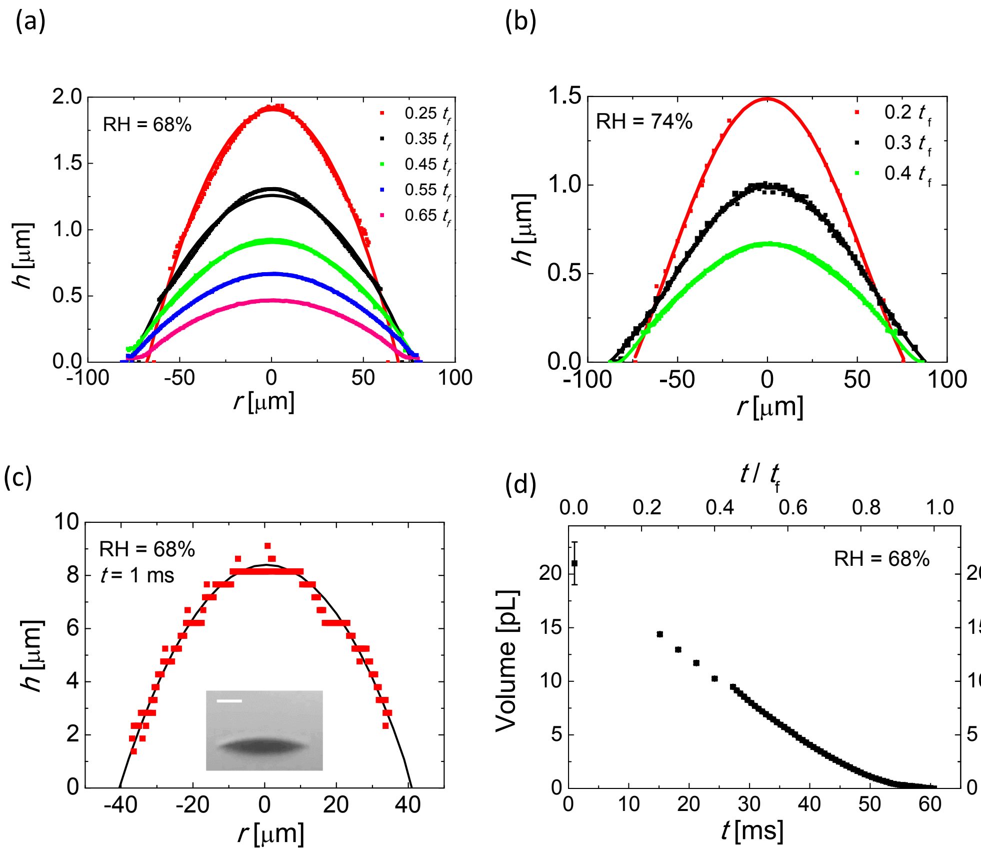

Fig. S1. Profiles for (a) RH = 68% and (b) RH = 74% at earlier times (see later times in Fig. 1b). Symbols are experimental data, and lines are (a) parabolic fit for $t \leq 0.45t_f$, and (b) 4th-order polynomial fit for $t = 0.2t_f$ and 9th-order polynomial fit to $t = 0.3t_f$ and $0.4t_f$. (c) Droplet profile from side-view image (inset) for RH = 68%. Symbols are experimental data, and the line is fit to a circular arc. Scalebar for inset image is 20 μm. (d) Droplet volume as a function of time for RH = 68%. The volume at t = 1 ms is obtained from a side-view image of a droplet (error bar shown) and the rest are from the reconstruction of the profile (see Methods).

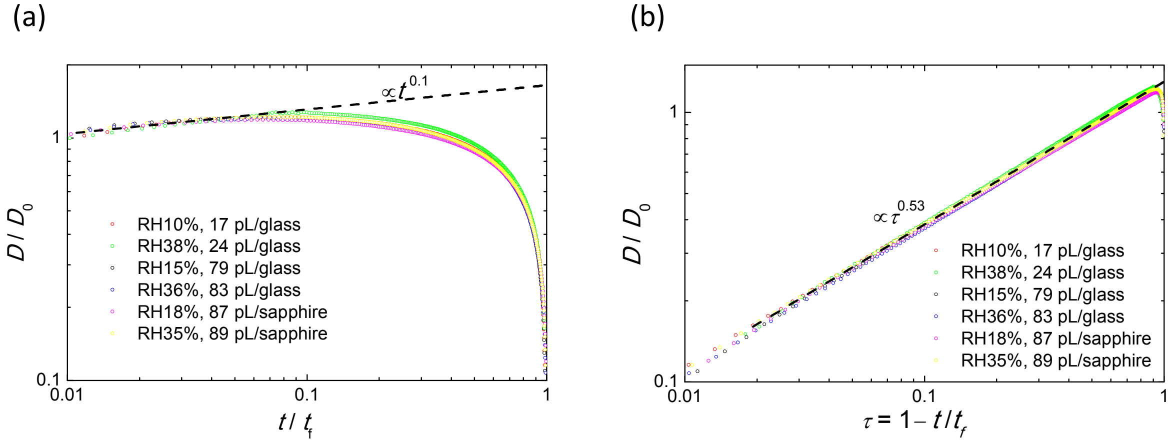

Fig. S2. (a) Normalised contact diameter as the function of normalised elapsed time for IPA at RH < 40% with a varied droplet volume and substrate. A 'universal' curve is obtained independent of droplet size (ca. 20 pL or 80 pL) and substrate (silica or sapphire). The time zero is defined as one frame before droplet impact. The contact diameter is normalised by D_0, where D_0 is the value in the frame immediately after the impact phase is over ($t \sim$ 1 ms), and the time by t_f. The spreading at early stage (0.01 t_f – 0.04 t_f) follows Tanner's law(12): the black dotted lines are the fit to $D \propto t^{0.1}$. (b) Contact diameter D as function of normalised remaining time $\tau = 1 - t/t_f$. Black dotted lines are a fit to $D \propto \tau^{0.53}$, in agreement with diffusion-controlled theory(13).

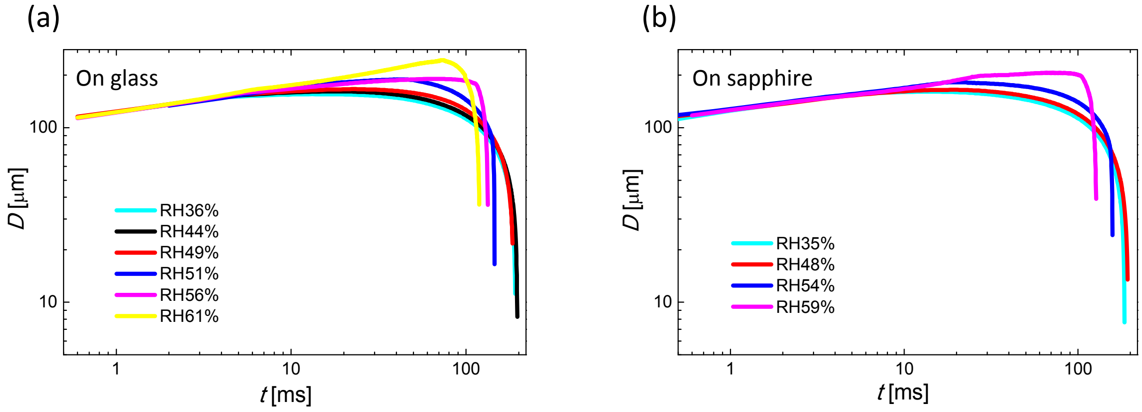

Fig. S3. Contact diameter of IPA sessile droplet as a function of the elapsed time at various RH, printed on (a) glass and (b) sapphire from a nozzle with orifice diameter of 50 μm. Initial droplet volume is in the range of (a) 72 – 83 pL, and (b) 87 – 91 pL.

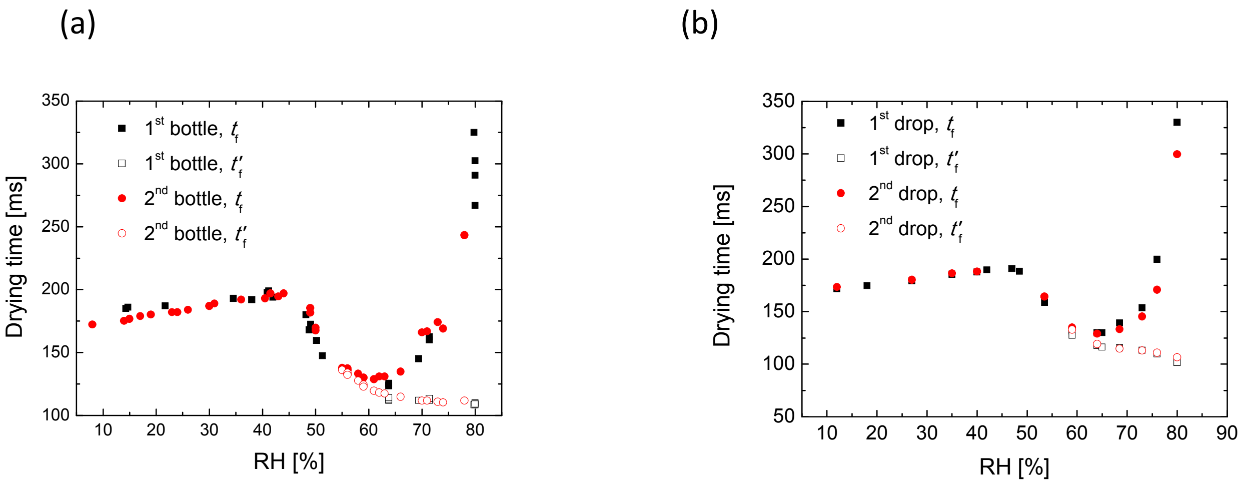

Fig. S4. Droplet lifetimes, t_f, and the lifetimes of the IPA-rich central cap, t'_f, as a function of RH. (a) Two independent measurements (with a two-month gap) on a glass substrate. Initial droplet volume is in the range of 72 – 83 pL. IPA in the first experiment was from an IPA bottle opened 15 days before the experiment. IPA in the second experiment was from a freshly opened bottle. (b) Data for two consecutively printed droplets at 2 Hz on a sapphire substrate. The 1st droplet is generated from a nozzle with an idle time of ~ 2 s after the previous drop is dispensed. The initial droplet volume is in the range of 87 – 91 pL.

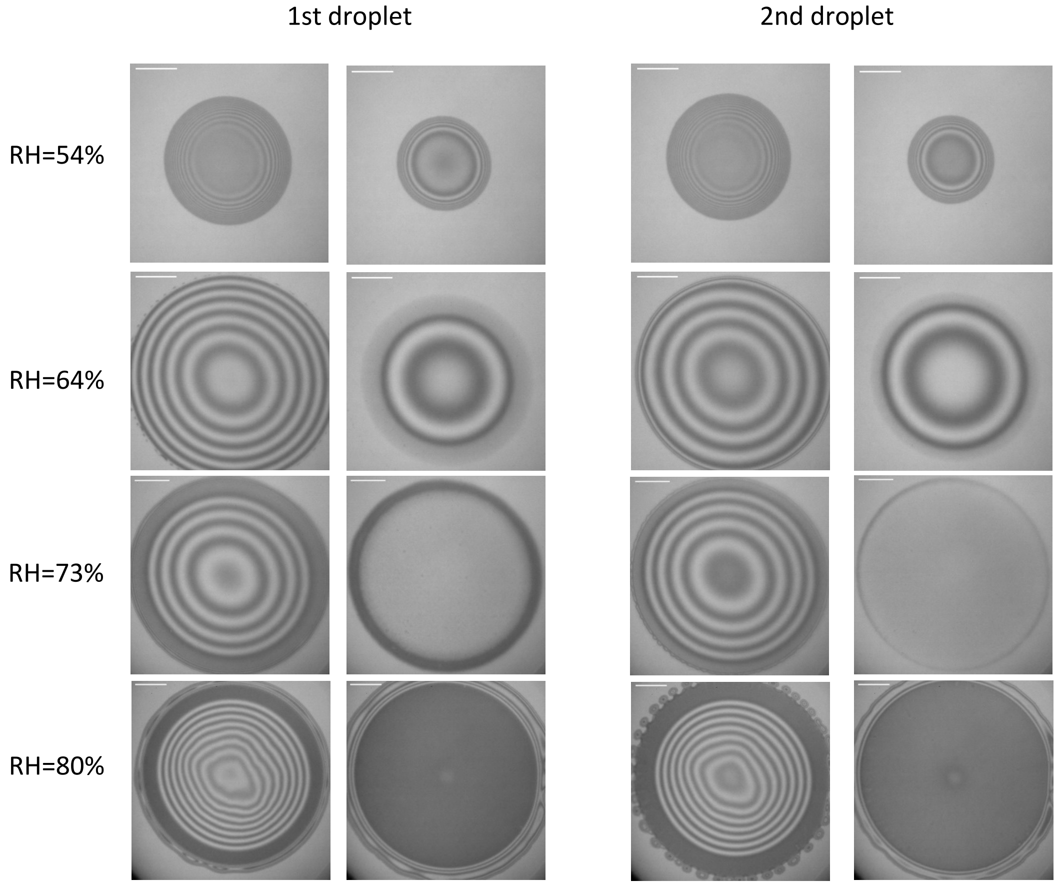

Fig. S5. Snapshots of droplet for two consecutively printed droplets at 2 Hz on sapphire substrate. RH = 54%, 64%, 73% and 80% from the top row down. The 1st droplet is generated from a nozzle with an idle time of ~ 2 s after the previous drop is dispensed. For each droplet, snapshots are taken at 0.5 t_f (left column) and 0.8 t_f (right column) for RH = 54%, 64%, 73%, and 0.5 and 1 t'_f for RH = 80%. The initial droplet volume is in the range of 87 – 91 pL. Scale bars: 50 μm. The only significant difference in the drying behaviour for the two delays before printing (0.5 s and 2s) is in the rim instability at RH = 80%. See also Supplementary Movie 10 for droplets drying on glass, showing a thinner rim and smaller wavelength, Λ, for the drop with a shorter idle time.

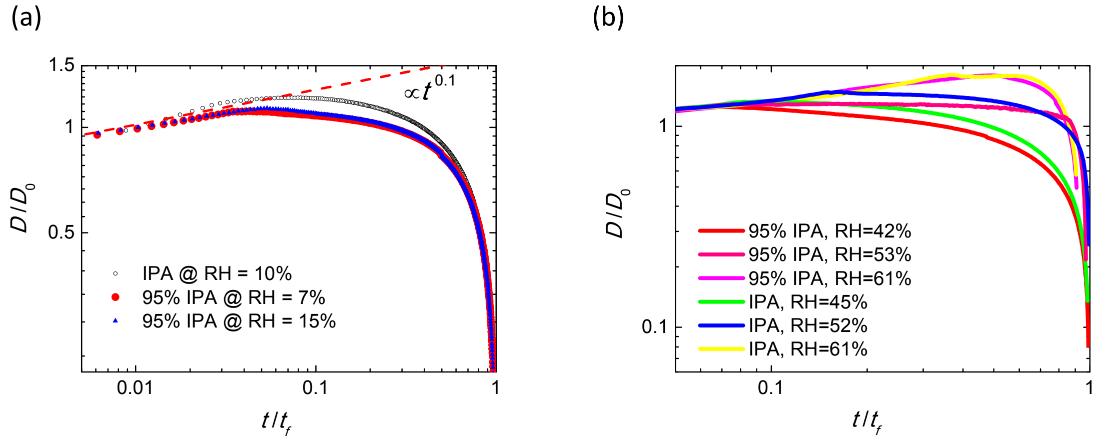

Fig. S6. Normalised contact diameter as the function of normalised elapsed time for a 95% IPA-water mixture in comparison with pure IPA, at (a) low RH, and (b) medium RH. The dotted red line in (a) is the fit to $D \propto t^{0.1}$.

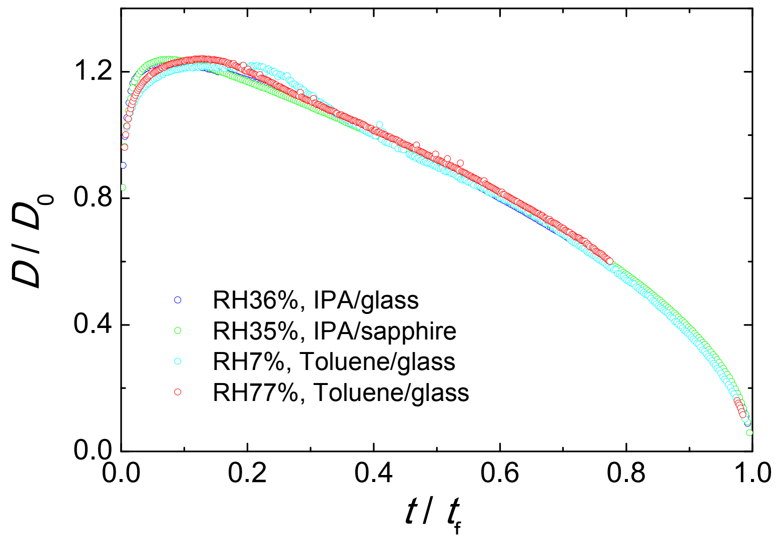

Fig. S7. Spreading and drying curves of toluene at RH = 7% and 77%, compared with IPA at 35-36% RH.

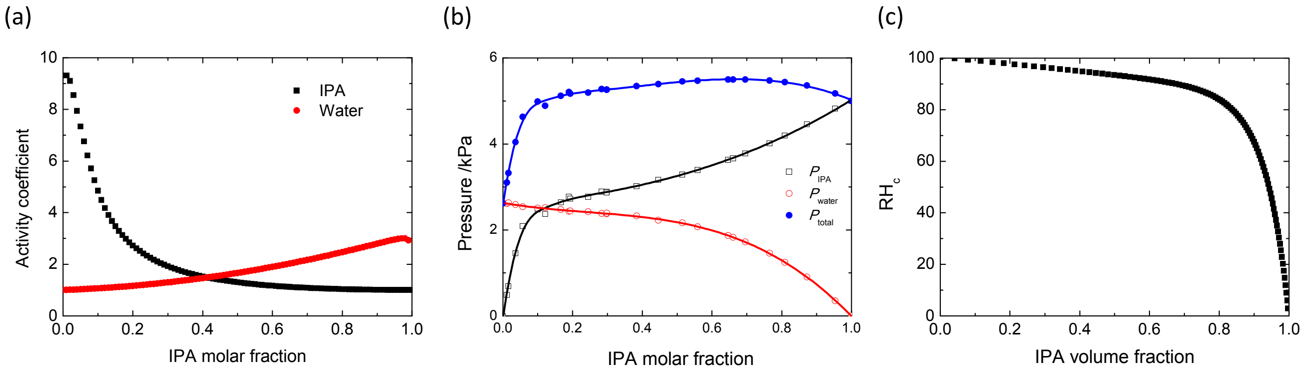

Fig. S8. (a) Activity coefficient as a function of IPA mole fraction (11). (b) Partial and total vapor pressure in IPA/water mixture as a function of IPA mole fraction. The total and partial pressures of IPA/water mixture calculated from activity coefficients in (a) are shown with symbols. The solid lines are B-spline interpolations. We assume IPA/water is a non-ideal liquid mixture but an ideal gas mixture so that the total pressure $p = p_1 + p_2 = x_1\Gamma_1 p_1^{sat} + x_2\Gamma_2 p_2^{sat}$, where subscript 1 refers to IPA and subscript 2 refers to water, x is the mole fraction for liquid phase and Γ is the activity coefficient. p^{sat} is derived from Antoine equation at 22 ℃(14). (c) The equilibrium volume fraction of IPA for different RH. This curve shows the critical RH, RH$_c$, above which water condenses: RH$_c = \Gamma_2 x_2$.

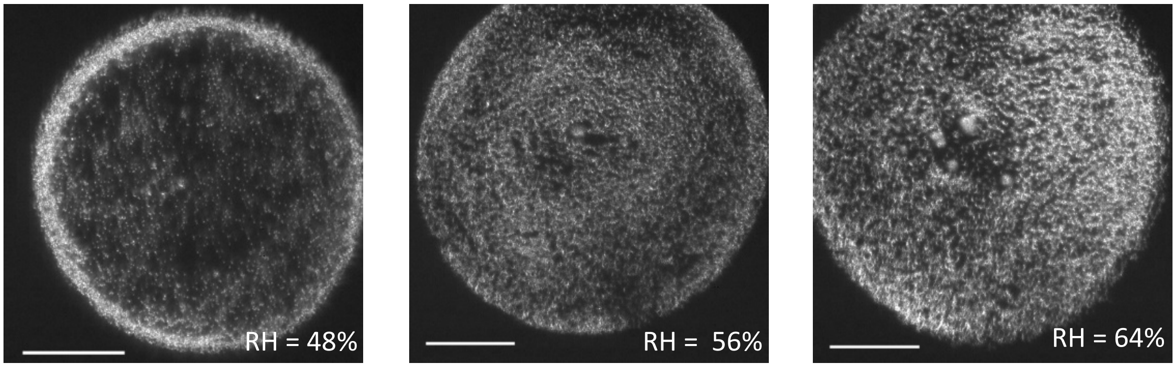

Fig. S9. Particle (755 nm polystyrene) deposit from IPA suspension on glass under the RH of (a) 48%, (b) 56% and (c) 64%. Scale bar is 50 μm. RH has a significant effect on the deposit with a pronounced ring at RH = 48% and a more uniform deposit at RH = 56% and 64%.

Movie S1. IPA droplet drying on glass under RH of 38%.

Movie S2. IPA droplet drying on glass under RH of 56%.

Movie S3. IPA droplet drying on glass under RH of 61%.

Movie S4. IPA droplet drying on glass under RH of 68%.

Movie S5. IPA droplet drying on glass under RH of 74%.

Movie S6. IPA droplet drying on glass under RH of 78%.

Movie S7. IPA droplet drying with trace particles on glass under RH of 46%.

Movie S8. IPA droplet drying with trace particles on glass under RH of 54%.

Movie S9. IPA droplet drying with trace particles on glass under RH of 64%.

Movie S10. Drying of two successive IPA droplets on glass under RH of 74% with idle times of 2 s for the first droplet and 0.5 s for the second droplet.

Frame rate for Movie 1 – 5 is 5000 fps. Scalebars are 30 μm. Droplets are generated from 30 μm nozzle. Frame rate for Movie 6 and Movie 10 are 5000 fps. Scalebars are 50 μm. Droplets are generated from 50 μm nozzle. Frame rate for Movie 7 – 9 is 1000 fps. Scalebars are 50 μm. Droplets are generated from 50 μm nozzle.

References

1. RH Perry, DW Green, JO Maloney, eds., *Perry's Chemical Engineers' Handbook*. (McGraw-Hill), p. 46 (1997).
2. AA Pahlavan, L Yang, CD Bain, HA Stone, Evaporation of Binary-Mixture Liquid Droplets : The Formation of Picoliter Pancakelike Shapes. *Phys. Rev. Lett.* **127**, 24501 (2021).
3. WM Haynes, ed., *CRC handbook of chemistry and physics*. (CRC press), (2011).
4. WD Ristenpart, PG Kim, C Domingues, J Wan, HA Stone, Influence of substrate conductivity on circulation reversal in evaporating drops. *Phys. Rev. Lett.* **99**, 1–4 (2007).
5. RG Larson, Transport and Deposition Patterns in Drying Sessile Droplets. *J Rev. Transp. phenomena fluid mechanics* **60**, 1538 (2014).
6. G Vázquez, E Alvarez, JM Navaza, Surface Tension of Alcohol + Water from 20 to 50 °C. *J Chem Eng Data* **40**, 611–614 (1995).
7. H Hu, RG Larson, Analysis of the effects of Marangoni Stresses on the microflow in an evaporating sessile droplet. *Langmuir* **21**, 3972 (2005).
8. P Chen, S Harmand, S Ouenzerfi, J Schiffler, Marangoni Flow Induced Evaporation Enhancement on Binary Sessile Drops. *J. Phys. Chem. B* **121**, 5824–5834 (2017).
9. W Pratt, K. C. Wakeham, The mutual diffusion coefficient for binary mixtures of water and the isomers of propanol. *Proc. Royal Soc. London. A. Math. Phys. Sci.* **342**, 401–419 (1975).
10. SK Parimalanathan, S Dehaeck, A Rednikov, P Colinet, Controlling the wetting and evaporation dynamics of non-ideal volatile binary solutions. *J. Colloid Interface Sci.* **592**, 319–328 (2021).
11. A Wilson, EL Simons, Vapor-Liquid Equilibria. *Ind. & Eng. Chem.* **44**, 2214–2219 (1952).

12. L Tanner, The spreading of silicone oil drops on horizontal surfaces. *J Phys. D: Appl Phys* **12**, 1473 (1979).
13. MA Saxton, JP Whiteley, D Vella, JM Oliver, On thin evaporating drops: When is the d2-law valid? *J. Fluid Mech.* **792**, 134–167 (2016).
14. C Yaws, ed., *Chemical Properties Handbook: Physical, Thermodynamics, Engironmental Transport, Safety & Health Related Properties for Organic & Inorganic Chemical [Hardcover].* (McGraw-Hill), p. 784 (1998).
